# Long‐Term Impact of EGUIDE Training on Facility‐Wide Guideline Adherence Rate in Schizophrenia and Major Depressive Disorder—A Methodological Reflection

**DOI:** 10.1002/npr2.70103

**Published:** 2026-02-25

**Authors:** Ghufran Saeed Rizvi

**Affiliations:** ^1^ Shifa College of Medicine Islamabad Pakistan

## Abstract

This Letter highlights crucial methodological flaws, including selection bias and ignored data clustering, in the EGUIDE training impact study. We recommend adopting quasiexperimental designs, multilevel modeling, and patient‐centered outcomes to ensure future research accurately confirms the program's true clinical effectiveness.

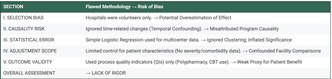

Dear Editor,

I read with great interest the article “Long‐Term Impact of EGUIDE Training on Facility‐Wide Guideline Adherence Rate in Schizophrenia and Major Depressive Disorder: A Multicenter Study” by Naomi Hasegawa et al., published in *Neuropsychopharmacology Reports* (DOI: 10.1002/npr2.70067). The study made an important contribution by examining how EGUIDE training improved psychiatric guideline adherence across Japan. The large sample size and multicenter approach are strengths of this work.

However, some methodological points need more attention. First, the study included only hospitals that volunteered for the EGUIDE program. Such hospitals may already have better facilities, more motivated staff, or higher awareness of guideline‐based care. This may have caused selection bias and overestimated the true impact of training. Studies by Millard et al. [1] and Enzenbach et al. [2] have shown that structured recruitment and baseline comparisons can reduce this bias. Including preparticipation data about hospitals would improve generalizability.

Second, the study design lacked quasiexperimental elements, such as interrupted time‐series or difference‐in‐differences analysis. These methods help to separate the program's effects from other national mental health initiatives or gradual improvements in care. Trutschel et al. [3] and Chang and Stuart [4] noted that ignoring time‐related changes in observational studies may lead to false causal conclusions.

Third, the authors used simple logistic regression, though the data were collected from multiple hospitals. This ignores the fact that results from the same facility are related. Multilevel models or generalized estimating equations, as suggested by Austin and Kapral [5], would account for clustering and provide more accurate results. These methods are now widely used in multicenter studies.

Fourth, the study adjusted only for limited factors such as age, sex, and hospital type. It did not control for disease severity, comorbidities, or illness duration, which can strongly influence outcomes. As shown by Sibert et al. [6] and Groenewegen et al. [7], adjusting for these case‐mix factors helps to make fair comparisons between hospitals.

Lastly, the quality indicators (QIs) used in this study mainly measured process outcomes such as polypharmacy reduction and use of CBT. These do not directly reflect patient benefits. Imani et al. [8] and Sterrantino et al. [9] recommend including both process and patient‐centered outcomes, such as relapse rate or quality of life, to capture the real clinical effect. The comprehensive summary of methodological issues and recommendations is presented in Table 1.

**TABLE 1:** Summary of methodological issues and recommendations.

Issue	Potential impact	Recommended improvement
Selection bias	Overestimation of EGUIDE impact	Include baseline comparisons and structured recruitment
Temporal confounding	Misattributed causality	Use interrupted time‐series or difference‐in‐differences designs
Ignored clustering	Inflated significance	Apply multilevel or hierarchical models
Limited case‐mix control	Confounded facility comparisons	Include illness severity and comorbidity adjustments
QI validity concerns	Weak proxy for actual patient benefit	Integrate patient‐centered and outcome‐based indicators

Future studies on educational programs like EGUIDE should include these methodological improvements. Careful selection of hospitals can reduce bias. Quasiexperimental designs can separate program effects from background trends. Multilevel analysis can improve accuracy. Broader covariate adjustment and better outcome measures can make findings more meaningful.

The EGUIDE program is a valuable national initiative for improving mental health care in Japan. Strengthening its research design will ensure that its benefits reflect real clinical improvements. With stronger methods and broader outcomes, EGUIDE can become a global model for evidence‐based psychiatric practice.

## Funding

The author has nothing to report.

## Ethics Statement

The author has nothing to report.

## Consent

Not required.

## Conflicts of Interest

The author declares no conflicts of interest.

## Data Availability

Data sharing not applicable to this article as no datasets were generated or analyzed during the current study.
